# A Magnetic Resonance Image Based Atlas of the Rabbit Brain for Automatic Parcellation

**DOI:** 10.1371/journal.pone.0067418

**Published:** 2013-07-02

**Authors:** Emma Muñoz-Moreno, Ariadna Arbat-Plana, Dafnis Batalle, Guadalupe Soria, Miriam Illa, Alberto Prats-Galino, Elisenda Eixarch, Eduard Gratacos

**Affiliations:** 1 Fetal and Perinatal Medicine Research Group, Institut d'Investigacions Biomediques August Pi i Sunyer (IDIBAPS), Barcelona, Spain; 2 Experimental 7T MRI Unit, Institut d'Investigacions Biomediques August Pi i Sunyer (IDIBAPS), Barcelona, Spain; 3 Maternal-Fetal Medicine Department, ICGON, Hospital Clínic, Universitat de Barcelona, Barcelona, Spain; 4 Laboratory of Surgical NeuroAnatomy (LSNA), Human Anatomy and Embryology Unit, Faculty of Medicine, Universitat de Barcelona, Barcelona, Spain; 5 Centro de Investigación Biomédica en Red de Enfermedades Raras (CIBERER), Barcelona, Spain; University of Salamanca- Institute for Neuroscience of Castille and Leon and Medical School, Spain

## Abstract

Rabbit brain has been used in several works for the analysis of neurodevelopment. However, there are not specific digital rabbit brain atlases that allow an automatic identification of brain regions, which is a crucial step for various neuroimage analyses, and, instead, manual delineation of areas of interest must be performed in order to evaluate a specific structure. For this reason, we propose an atlas of the rabbit brain based on magnetic resonance imaging, including both structural and diffusion weighted, that can be used for the automatic parcellation of the rabbit brain. Ten individual atlases, as well as an average template and probabilistic maps of the anatomical regions were built. In addition, an example of automatic segmentation based on this atlas is described.

## Introduction

Animal models are essential for the understanding of brain and neurodevelopment. Several species have been used in neuroscience research, from primates to small animals such as rat, mouse and rabbit. The rabbit has been widely employed for modeling brain damage after perinatal injury in humans because it presents a human-like timing of perinatal brain white matter maturation [1]. Rabbit models of intrauterine inflammation [2], cerebral palsy [1], [3] and intrauterine growth restriction [4] have been developed, demonstrating changes in neonatal neurobehavior and in brain structure [1]–[6].

Brain atlases have become an essential tool for the analysis of structural and functional differences in neuroimage, allowing volume and shape quantification of brain regions, for mapping functional activation and connectivity analysis. Over recent years, traditional 2D histological based atlases have been complemented by the generation of 3D digital atlases based on different image modalities, especially in magnetic resonance image (MRI). Although MRI-based atlases have less resolution than histological atlases, they present other advantages. Thus, 3D acquisition allows the volumetric reconstruction of brain regions, preserving the spatial relationship within the brain. Moreover, the digital format allows the application of image processing algorithms for quantification or automatic segmentation as well as comparisons between different subject acquisitions. Digital brain atlases have been developed for a number of species used in research, including mouse [7]–[12], rat [13], [14], canary [15] or monkey [16]–[19].

However, to the best of our knowledge, there is no digital rabbit brain atlas available in the literature. MRI studies using the rabbit as a model have been based on manual delineation of the areas of interest. For this reason, we developed an MRI-based atlas for the New-Zealand rabbit brain, suitable for automatic segmentation. Delineation of regions was performed taking into account both T1-weighted and diffusion MRI, based on regions defined by histological atlases [20], [21]. Nevertheless, some of the smaller regions described in these atlases, which cannot be identified radiologically, were not included in the template.

The brain region delineation as well as the brain template and the probabilistic atlas is available on-line in www.medicinafetalbarcelona.org/rabbitbrainatlas, where the brain parcellation can be visualized and downloaded in order to be used for automatic segmentation.

## Materials and Methods

In order to build the radiological rabbit brain atlas, T1 and diffusion MRI volumes of a set of 10 healthy adult rabbits were acquired and radiologically identifiable regions were manually delineated in these subjects. As a result, 10 individual brain atlases were obtained. Based on the 10 acquisitions, a brain template representing the average shape and intensity of T1-MRI brain volumes was built and a probabilistic atlas was developed, which defines at each point the probability of belonging to a specific region. Each of these steps are deeper described above.

### Data

The atlas was constructed on a set of 10 healthy adult control New Zealand rabbits at 70 post-natal days (weight 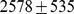 g, 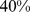 male, 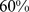 female). An additional healthy adult rabbit was used to test the performance of the region segmentation based on the atlas developed on the 10 experimental subjects. Animal experimentation of this study was approved by the Animal Experimental Ethics Committee of the University of Barcelona (permit number: 206/10–5440). Animal handling and all the procedures were performed following all applicable regulations and guidelines of the Animal Experimental Ethics Committee of the University of Barcelona. Included rabbits were obtained by Cesarean section at 30 days of gestation from New Zealand pregnant rabbits provided by a certified breeder. Rabbits were housed by a wet nurse rabbit until 30th postnatal day when they were weaned. Then, rabbits were housed in groups of three on a reversed 12/12 h light cycle with free access to water and standard chow. At 70th postnatal day, rabbits were anesthetized with ketamine 35 mg/kg and xylazine 5 mg/kg given intramuscularly and were sacrificed with an overdose of sodium pentobarbital (200 mg/kg) endovenous injection. Left and right common carotid arteries were cannulated and brains were perfused with phosphate-buffered saline (PBS) followed by 4% paraformaldehyde PBS. Finally, brains were dissected and fixed in 4% paraformaldehyde PBS at 4°C for 48 h.

The acquisition was performed on the excised and fixed brain using a 7 T animal MRI scanner (Bruker BioSpin MRI GMBH). High-resolution three-dimensional T1 weighted images were obtained by a Modified Driven Equilibrium Fourier Transform (MDEFT) 3D sequence with the following parameters: echo time (TE) = 3.5 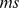, repetition time (TR) = 4000 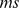, slice thickness = 0.7 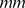 with no interslice gap, 70 coronal slices and in-plane acquisition matrix of 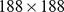, resulting in a voxel dimension of 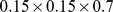 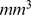.

For diffusion weighted images (DWI), Spin Echo DTI sequence was used to gain image quality, avoiding the artifacts associated to Echo Planar Imaging, but increasing the acquisition time [22]. Diffusion sensitizing gradients were applied along 126 directions with a 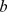-value of 3000 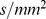, and a reference (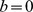) image was acquired. Other experimental parameters were: TE = 26 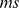, TR = 250 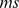, slice thickness = 0.7 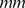 with no interslice gap, 70 coronal slices and in-plane acquisition matrix of 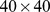, with a voxel dimension of 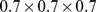 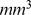.

### Image Processing

Previous to the manual delineation of the brain regions, image processing is required in order to take advantage of both T1 and diffusion MRI. The volumes acquired by both modalities were aligned, so T1 intensity and fiber orientation images can be jointly visualized to perform the delineation. Since these modalities have different resolution, a multimodal registration algorithm was applied to align both images. Registration based on the optimization of mutual information [23] between T1 and the baseline volumes of the diffusion protocol was implemented. The affine transformation estimated by the registration algorithm was applied to the diffusion images, and afterwards the tensor image was estimated from the registered diffusion data set. The diffusion gradient direction is described with respect to the original image orientation. Consequently, changes in orientation due to the transformation applied to the diffusion images were also applied to the gradient direction [24].

In order to segment the brain from the background a mask was computed, by means of the Otsu threshold method [25]. Finally, the tensor at each voxel inside the mask was estimated using the least squares method described by [26].

Once the diffusion tensor image was computed, eigenanalysis was performed at each voxel. From eigenvalues, fractional anisotropy (FA) was computed and the first eigenvector was considered as the fiber direction [27]. Thus, the FA-color map, where color is related to fiber direction and intensity is weighted by FA was obtained.

### Regions Definition

Taking as gold standard reference the histological rabbit brain atlas [20], manual delineation of brain regions was performed on T1-weighted images overlaid with FA-color maps. In addition, mouse and rat atlases [28]–[32] were used as second reference when structures were not described in rabbit atlas.

Every brain structure was firstly delineated in the plane where was more clearly identifiable, and then corrected in the other two orthogonal planes. Although most of the regions were better identified in the coronal view, other planes were preferred for structures such as several cortical regions and the cerebellar vermis and hemispheres.

**Figure 1:**
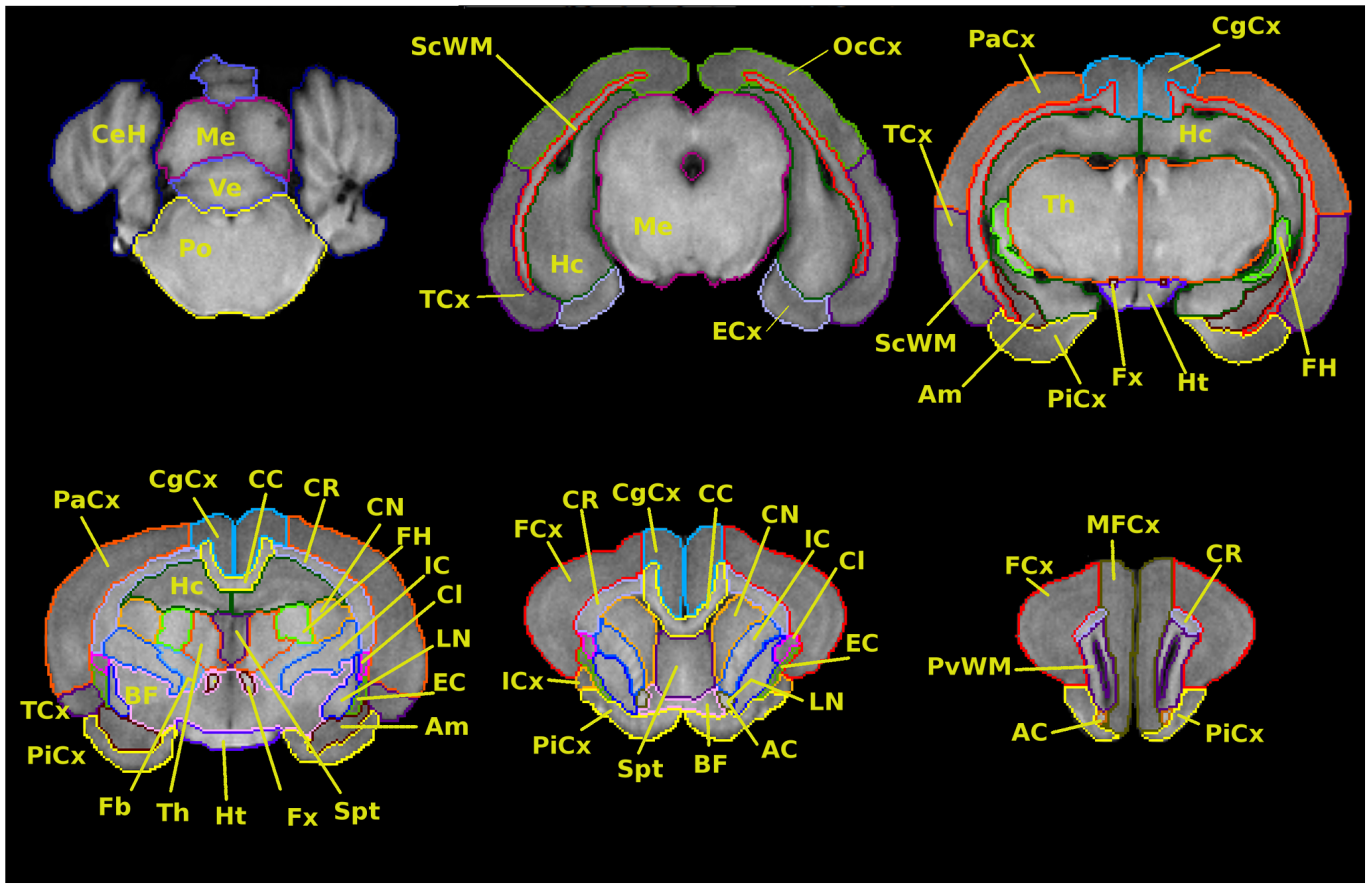
Anatomical regions delineated over the T1 images. Cerebellar hemispheres (CeH), mesencephalon (Me), vermis (Ve), pons (Po); subcortical white matter (Sc WM), hippocampus (Hc), entorhinal cortex (ECx), temporal cortex (TCx), occipital cortex (OcCx); piriform cortex (PiCx), parietal cortex (PaCx), cingulate cortex (CgCx), fimbria of hippocampus (FH), fornix (Fx), thalamus (Th), hypothalamus (Ht), amygdala (Am); external capsule (EC), internal capsule (IC), claustrum (Cl), lenticular nucleus (LN), caudate nucleus (CN), corona radiata (CR), corpus callosum (CC), septum (Spt), forebrain (Fb), basal forebrain (BF); frontal cortex (Fcx), insular cortex (Icx), anterior commissure (AC); medial frontal cortex (MFCx), periventricular white matter (PvWM).

An example of the delineation of brain regions over representative slices of T1-weighted images is displayed in Figure 1. Furthermore, in the results section, the T1 intensity values and diffusion parameters characterizing each structure were compiled.

60 brain regions were defined, considering left and right structures separately when appropriate, which were classified into four groups: cortical regions, white matter (WM), deep gray matter (GM) and “other regions”:

- Cortical regions: frontal, medial frontal, cingulate, piriform, entorhinal, parietal, occipital, insular and temporal cortex.
- White matter: periventricular white matter, internal and external capsule, corona radiata, fimbria of hippocampus, fornix, subcortical white matter, corpus callosum and anterior commissure.
- Deep gray matter: claustrum, caudate nucleus, amygdala, thalamus, hypothalamus, hippocampus, lenticular nucleus and olfactory bulb.
- “Other regions”: cerebellar hemispheres, vermis, forebrain, basal forebrain, diencephalon, mesencephalon, pons, medulla oblongata and septum.

Note that region definition was based on radiological acquisitions, and therefore, finer regions requiring histological criteria to be identified are not included in the atlas. Without the aim of fully describe the delineated regions, below we include some guidelines taken into account to define the limits of certain structures, specially those structures that we have adapted from other species' brain atlases.

Regarding cortical regions, since no continuous cortical parcellation is available in the New Zealand’s histological rabbit brain atlas [20], the delineation of most cortical areas, namely frontal, occipital, temporal, parietal, insular, piriform and entorhinal cortices, was performed based on mouse and/or rat atlases [28]–[32]. From anterior to posterior the cortical regions were labeled as follows: the medial portion of the cortex was defined as medial frontal cortex until the appearance of corpus callosum, after which was labeled as cingulate cortex. Frontal cortex region included the lateral parts of the cortex containing motor and sensory-motor areas [32]. The ventral part of the cortex was divided in olfactory, piriform and entorhinal cortices. Thus, following anterio-posterior direction, olfactory cortex was upper-limited by rhinal fissure. When rhinal fissure was not distinguishable, it became piriform cortex, which continued until the starting of the amygdala, where the beginning of entorhinal cortex was defined [28].

The delineation of WM regions was based on the work of Shek et al. [20]. Following anterior-posterior direction, we first found periventricular WM, which was considered as the WM surrounding lateral ventricles until the presence of the genus of the corpus callosum. Corona radiata, external and internal capsules were present also in the most anterior slices. When these structures, together with corpus callosum became not visible, subcortical WM is defined, until the end of WM bundles in the posterior part.

With regards to the GM regions, their delineation was based in the histological rabbit brain atlas [20] except for the amygdala, that was based in a rat atlas [28]. Namely, amygdala was identified as the GM region surrounded by the posterior limit of the insular cortex and the anterior limit of entorhinal cortex. On the other hand, the anterior limit of the thalamic region coincides with the most anterior part of the fimbria of hippocampus. Thalamic region enclosed main thalamic and habenular nuclei, that would required histological analysis to be properly identified. The posterior limit of thalamic region was identified by the appearance of the superior colliculus. Hippocampus was easily identified as a multiple cortical layer structure in the coronal view and it included the hippocampal formation.

Finally, “other regions” category contained structures that did not fit in the previously define categories. This is the case of anatomical regions as cerebellar hemispheres, vermis, pons, medulla oblongata and septum and remainders of other brain regions as forebrain, basal forebrain, mesencephalon and diencephalon.

### Delineation

The software used for delineation was ITK-SNAP [33]. It allows overlay of different images, with different transparency levels, and therefore delineation can be based on different image modalities. As aforementioned, both T1-weighted and diffusion magnetic resonance images were considered for a more accurate identification of the different structures composing the white and gray matter.

In order to simplify the delineation procedure, once the first image is delineated, its parcellation is propagated to the second subject by an elastic registration, so it can be taken as a starting point of the manual delineation of this volume as reported in [10]. This procedure is repeated iteratively to parcel the 10 subjects. At each step, all the previous delineations were considered, so a better starting point for the manual delineation is obtained.

Therefore, let be 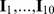 the ten images to be parcelled and 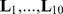 the label maps corresponding to the parcellation of each of the subjects. Manual delineation of the the first volume 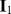 resulted in a label map 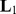. Subsequently, every brain volume 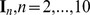 was segmented based on the previous label maps 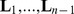, as follows:

1. The 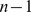 label maps previously obtained by manual delineation (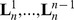) were propagated to volume 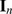 using an elastic registration algorithm. Thus, a set of 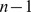 label maps 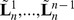 aligned to the volume 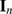 were estimated.
2. A label map of subject 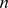, 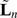, was computed combining 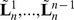, assigning to each voxel 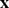 the most frequent label, that is:
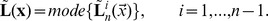

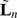
 is used as a starting point for the manual delineation of the brain regions of subject 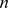, that results in 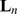.

This methodology resulted in a set of 10 individual atlases, that is, the region parcellation of the 10 brain volumes.

### Average Template

A population template was built, describing the average shape and intensities of a normal healthy brain. The procedure followed to obtain this template was similar to the described in [34], first the average shape template is estimated iteratively, and afterwards the mean intensity model is computed:

1. Let be 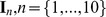 the ten volumes of healthy brains that were considered.
2. The most *normal* volume, 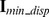 in the data set was chosen to initialize the iterative algorithm. It is defined as the volume requiring the minimum transformation to match all the other volumes in the dataset. The elastic transformation matching every pair of volumes was estimated by means of a block matching registration algorithm [35], resulting in a displacement vector field for each pair of images. For each of these transformations the mean displacement was computed. Finally, the image minimizing the mean displacement was used to initialize the iterative algorithm followed to determine the average shape template.
3. Once the minimum displacement image was identified, the mean shape, 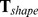 was computed by means of an iterative procedure. Let be 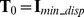 the initial estimation. It was registered against all the images 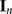 in the dataset, and the average transformation 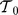 was computed. This transformation was applied to the current template to obtain the template for the next iteration 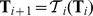. This procedure was repeated until the average transformation was smaller than a given threshold. In practice, convergence was achieved in few iterations. The mean shape template was obtained as 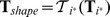, where 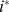 is the iteration in which convergence is reached.
4. Taking into account the mean shape, the average intensity volume was computed. That is, all the volumes were registered to the mean shape and the average intensity value at each voxel was computed. Voxels whose intensity was above two standard deviation of the mean value were excluded to avoid the effect of noise or misregistration in the template.

### Probabilistic Atlas

A probabilistic atlas was built over the template based on the 10 individual atlases, describing at any location the probability to belong to any of the regions.

The label maps 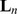, 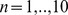 that had been delineated over each volume 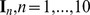 were propagated to the average template, resulting in a set of ten label maps 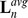. At each voxel 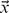 of the average template, the probability of belonging to a given region 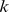 was estimated as
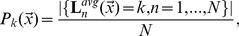
(1)where 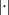 denotes the cardinal of the set, and 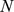 is the number of volumes considered to build the atlas, that is, 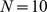. Thus, we obtained a set of probabilistic maps, one for each anatomical region delineated in the atlas. The use of this probabilistic approach is more robust against volume partial effect, since voxels in the edge between two regions (let be 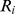 and 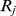) will have a certain probability 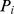 to belong to 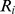 and a probability 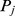 to belong to 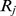, which is especially useful for the automatic parcellation.

Also a label map can be estimated on the template assigning to each voxel the label of the most probable region.

### Automatic Parcellation

The atlas can be used for automatic brain parcellation based on registration. Let be 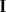 a new brain volume, segmentation is obtained by registering the template 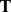 against it, assessing in that way the elastic transformation 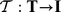. This transformation can be estimated by any of the software available for image registration. Applying this transformation to the region probability maps, the probability of a voxel in the image 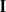 to belong to each of the regions is computed. Finally, each voxel is assigned to the region of maximum probability. It is also feasible to apply the transformation to the label map defined over the average template, obtaining in such way the label map in the new brain, although it could be less accurate than the probabilistic approach.

On the other hand, the accuracy of the segmentation relies on the performance of the registration algorithm. To test the approach, a multiresolution block-matching algorithm was implemented to perform registration, based on the correlation coefficient between T1-images [35]. The performance of segmentation is tested in the additional subject that was not included in the atlas building, and evaluated both qualitatively, by visual inspection, and quantitatively, by comparing with manual delineations performed by two different observers. Namely, Dice coefficients and confusion matrix [36] were used to measure the similarity between manual and automatic segmentation. The overlapping between two different parcellations was estimated by the Dice coefficient for each region 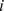:
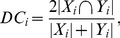
(2)were 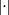 is the cardinal of the set, 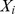 are the points that were labeled as belonging to region 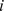 by the first parcellation being compared and 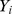 the points assigned to region 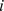 by the second parcellation. This index is computed to measure the similarity between automatic and manual delineation, as well as between both manual delineations. High and similar values of Dice coefficient in both cases will show the reliability of the automatic segmentation, meaning that differences are comparable to the interobserver variability.

In addition, a measure of the global matching was estimated as:
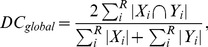
(3)


Besides, confusion matrix, measuring the percentage of voxels belonging to region 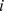 that have been labeled as region 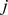, was built.

Obviously, automatic segmentation can be performed with other registration algorithms [37], and the accuracy of the result will rely on the performance of the registration algorithm.

## Results

### Anatomical Regions

**Figure 2:**
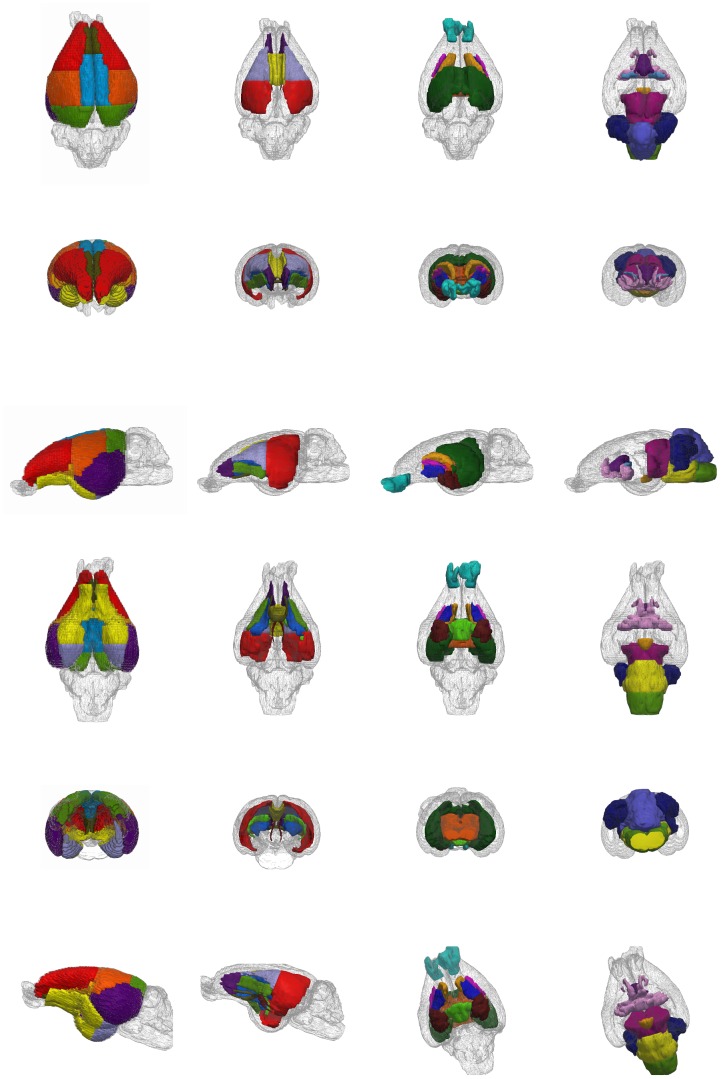
3D reconstruction of the brain regions of the rabbit. From left to right: cortical regions, white matter regions, deep gray matter, other regions. From top to bottom: dorsal view; anterior view; lateral view; ventral view; posterior view; and oblique view.

**Figure 3:**
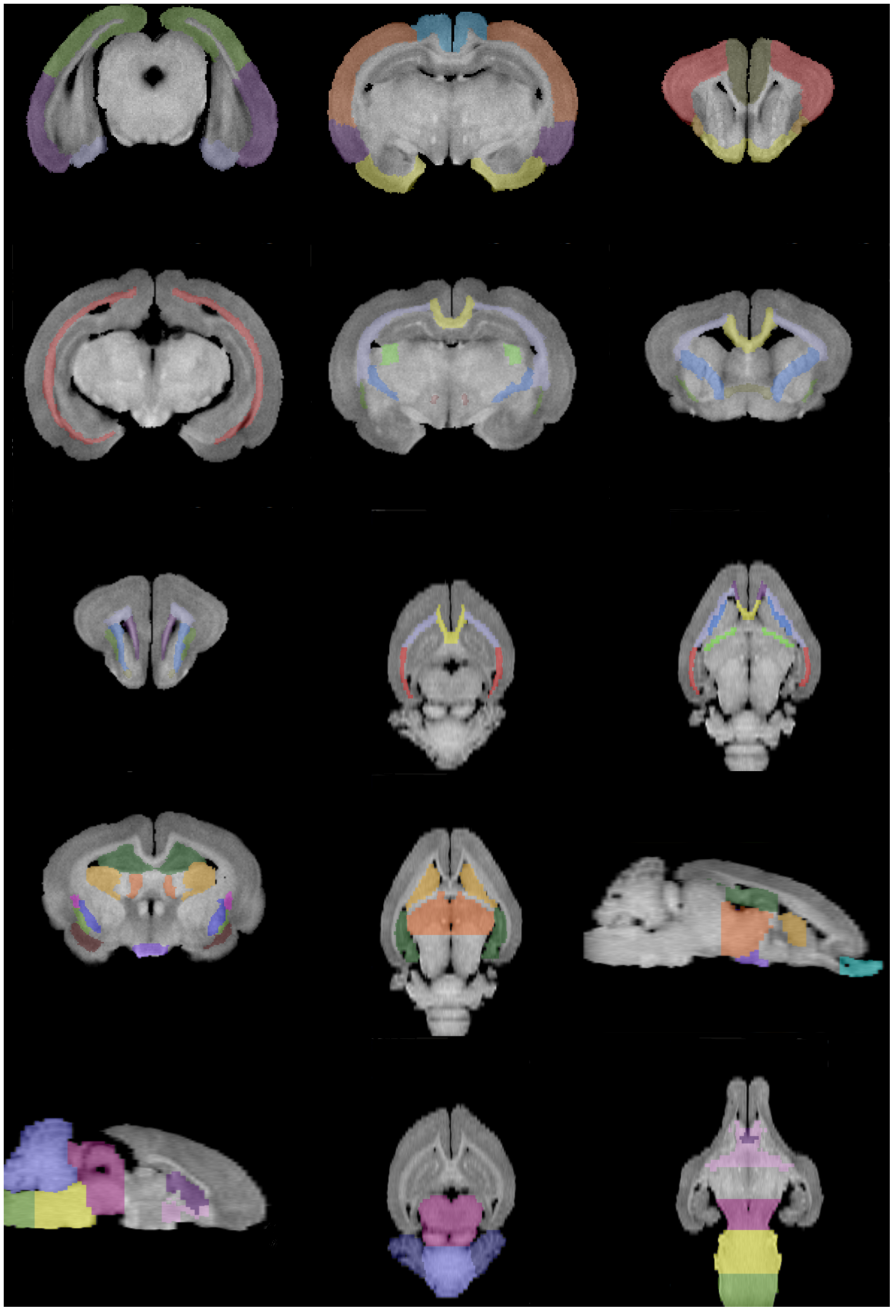
Brain regions overlapped over representative slices of the T1-weighted image. First row: cortical regions; second and third rows: white matter regions; fourth row: deep gray matter and fifth row: other regions.

As previously described, a set of 60 brain regions was defined for each volume. Each region was assigned to one of the following areas: cortical, white matter, deep gray matter, and “other regions”. Illustrative views of the 3D reconstruction of the regions included in the four main areas are depicted in Figure 2. Figure 3 displays representative slices of the T1-images, where the corresponding regions are overlapped for each major area. The properties of the structures, such as T1 intensity and diffusion parameters, are compiled in Table 1, as described below.

**Table 1:** Brain region characterization.

Region	Volume(%)	T1-intensity	FA	MD
Frontal cortex	7.89 (0.65)	0.93 (0.05)	0.18 (0.06)	1.00 (0.20)
Medial frontal cortex	2.45 (0.09)	0.94 (0.08)	0.18 (0.04)	1.07 (0.09)
Cingulate cortex	2.86 (0.30)	0.83 (0.06)	0.15 (0.04)	1.08 (0.06)
Piriform cortex	3.77 (0.19)	0.87 (0.04)	0.19 (0.04)	1.03 (0.14)
Entorhinal cortex	1.40 (0.09)	0.75 (0.05)	0.15 (0.02)	1.15 (0.12)
Parietal cortex	5.59 (0.40)	0.86 (0.02)	0.16 (0.03)	1.07 (0.06)
Occipital cortex	3.35 (0.23)	0.80 (0.03)	0.15 (0.04)	1.16 (0.11)
Insular cortex	0.66 (0.08)	0.86 (0.07)	0.21 (0.07)	1.06 (0.17)
Temporal cortex	5.92 (0.24)	0.82 (0.04)	0.13 (0.02)	1.08 (0.05)
External capsule	0.43 (0.04)	0.95 (0.08)	0.28 (0.03)	1.07 (0.06)
Internal capsule	1.49 (0.09)	1.28 (0.07)	0.35 (0.05)	0.97 (0.08)
Corpus callosum	0.70 (0.09)	1.15 (0.03)	0.24 (0.04)	1.04 (0.09)
Anterior commissure	0.13 (0.02)	1.30 (0.05)	0.27 (0.04)	0.96 (0.09)
Periventricular WM	0.65 (0.03)	1.16 (0.06)	0.26 (0.04)	1.04 (0.07)
Subcortical WM	2.82 (0.33)	1.04 (0.03)	0.21 (0.03)	1.04 (0.06)
Corona radiata	1.59 (0.13)	1.17 (0.05)	0.23 (0.06)	1.06 (0.09)
Fimbria of hippocampus	0.39 (0.06)	1.22 (0.05)	0.31 (0.14)	1.01 (0.08)
Fornix	0.07 (0.01)	1.28 (0.04)	0.22 (0.03)	0.92 (0.08)
Claustrum	0.17 (0.02)	0.99 (0.06)	0.32 (0.08)	1.01 (0.10)
Caudate nucleus	1.43 (0.11)	1.08 (0.03)	0.23 (0.05)	1.07 (0.05)
Thalamus	6.86 (0.39)	1.21 (0.05)	0.23 (0.02)	0.97 (0.03)
Hippocampus	9.39 (0.32)	0.92 (0.02)	0.19 (0.02)	1.07 (0.03)
Amygdala	1.26 (0.11)	0.98 (0.03)	0.21 (0.06)	1.00 (0.04)
Hypothalamus	0.67 (0.08)	1.09 (0.04)	0.20 (0.04)	0.96 (0.13)
Lenticular nucleus	0.69 (0.07)	1.18 (0.05)	0.30 (0.07)	1.03 (0.12)
Olfactory bulb	1.59 (0.41)	0.76 (0.05)	0.12 (0.06)	0.61 (0.03)
Cerebellar hemispheres	6.47 (0.82)	0.85 (0.02)	0.11 (0.03)	0.95 (0.13)
Vermis	8.08 (0.57)	1.02 (0.04)	0.14 (0.01)	0.92 (0.10)
Basal forebrain	1.65 (0.16)	1.13 (0.03)	0.26 (0.04)	0.95 (0.07)
Forebrain	0.23 (0.02)	1.19 (0.03)	0.28 (0.05)	0.94 (0.11)
Diencephalon	0.17 (0.03)	1.11 (0.11)	0.18 (0.05)	1.15 (0.45)
Mesencephalon	8.17 (0.54)	1.14 (0.03)	0.19 (0.01)	0.96 (0.05)
Pons	5.94 (0.33)	1.25 (0.06)	0.22 (0.01)	0.98 (0.11)
Medulla oblongata	4.13 (0.53)	1.17 (0.05)	0.18 (0.03)	0.78 (0.08)
Septum	0.88 (0.06)	1.07 (0.03)	0.23 (0.02)	1.02 (0.06)

Mean and standard deviation of region volume (corrected by total brain volume), relative T1-MRI intensity value, fractional anisotropy and relative mean diffusivity value in the study group.

### Individual Atlases

**Figure 4:**
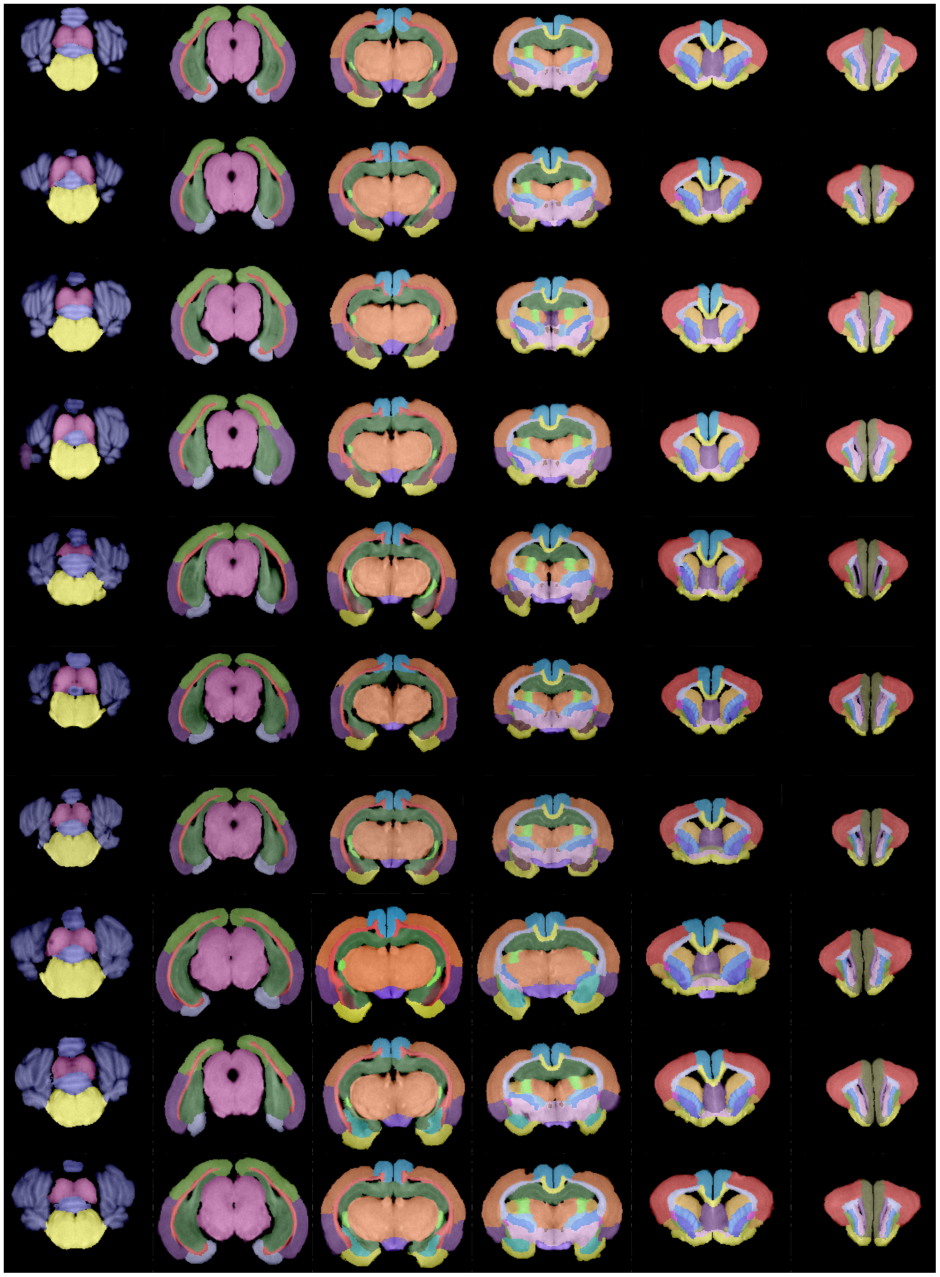
Individual atlases. Each row corresponds to one subject.

Ten individual atlases were developed as described in the Methods section. In Figure 4, different slices of the individual atlases are shown. Each row corresponds to a different subject and each column represents equivalent slices for each of the subjects, containing similar structures.

Different regions are characterized in Table 1, where mean values of regional volume (corrected for total brain volume) and relative regional T1 intensity (normalized by average T1 intensity in the whole brain) of brain regions are displayed, together with the mean values of regional fractional anisotropy (FA) and regional mean diffusivity (MD) normalized by average MD value in the whole brain. Thus, relative values will be higher than 1 if they are higher than the mean value in the whole brain, and lower than 1 if they are lower than the mean value.

Note that separate left and right sides of most bilateral structures had been taken as different regions in the atlas, obtaining 60 regions. However, for the sake of simplicity, in Table 1, left and right sides were considered altogether, resulting in 35 different regions.

### Template and Probabilistic Maps

**Figure 5:**
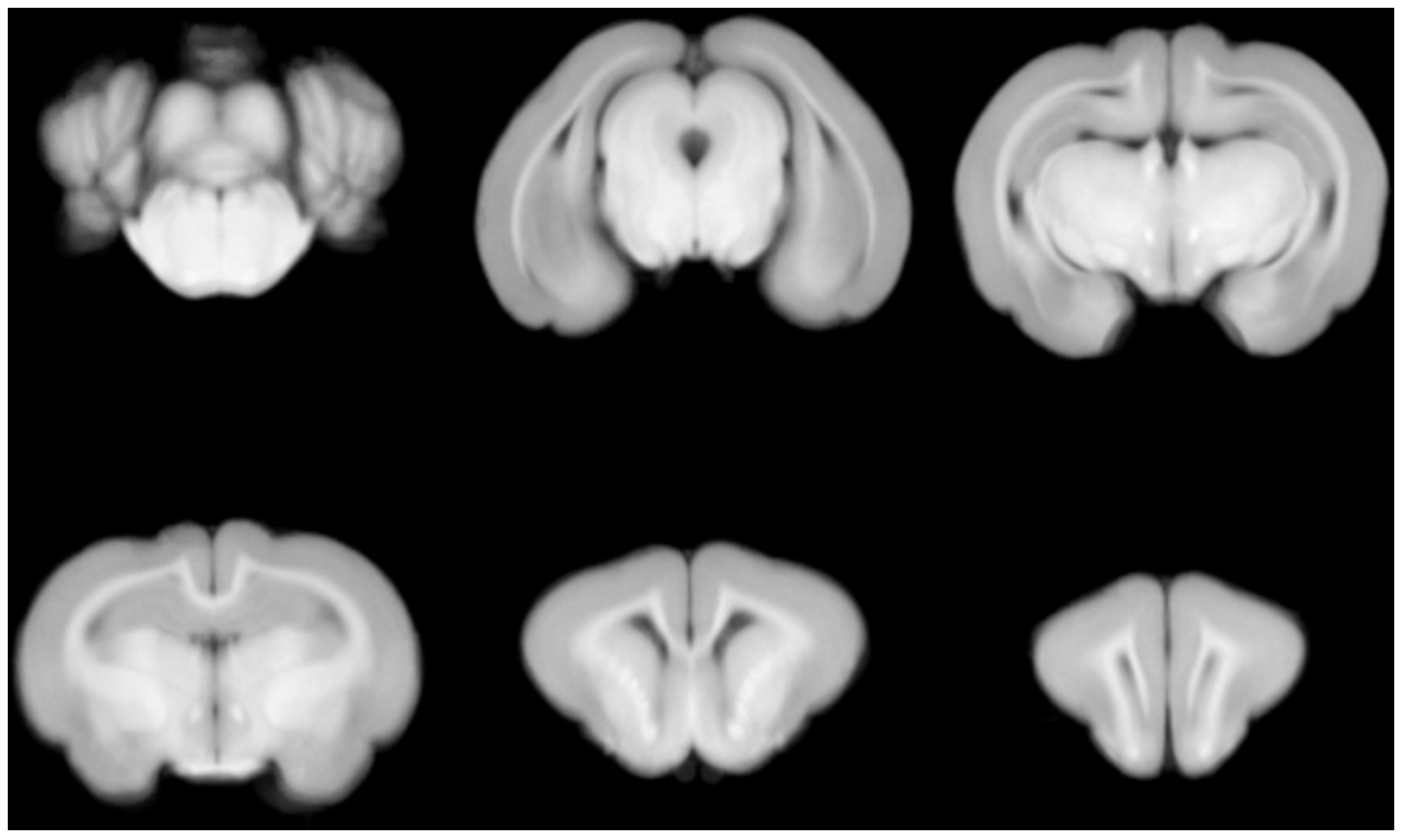
Representative slices of the average template.

**Figure 6:**
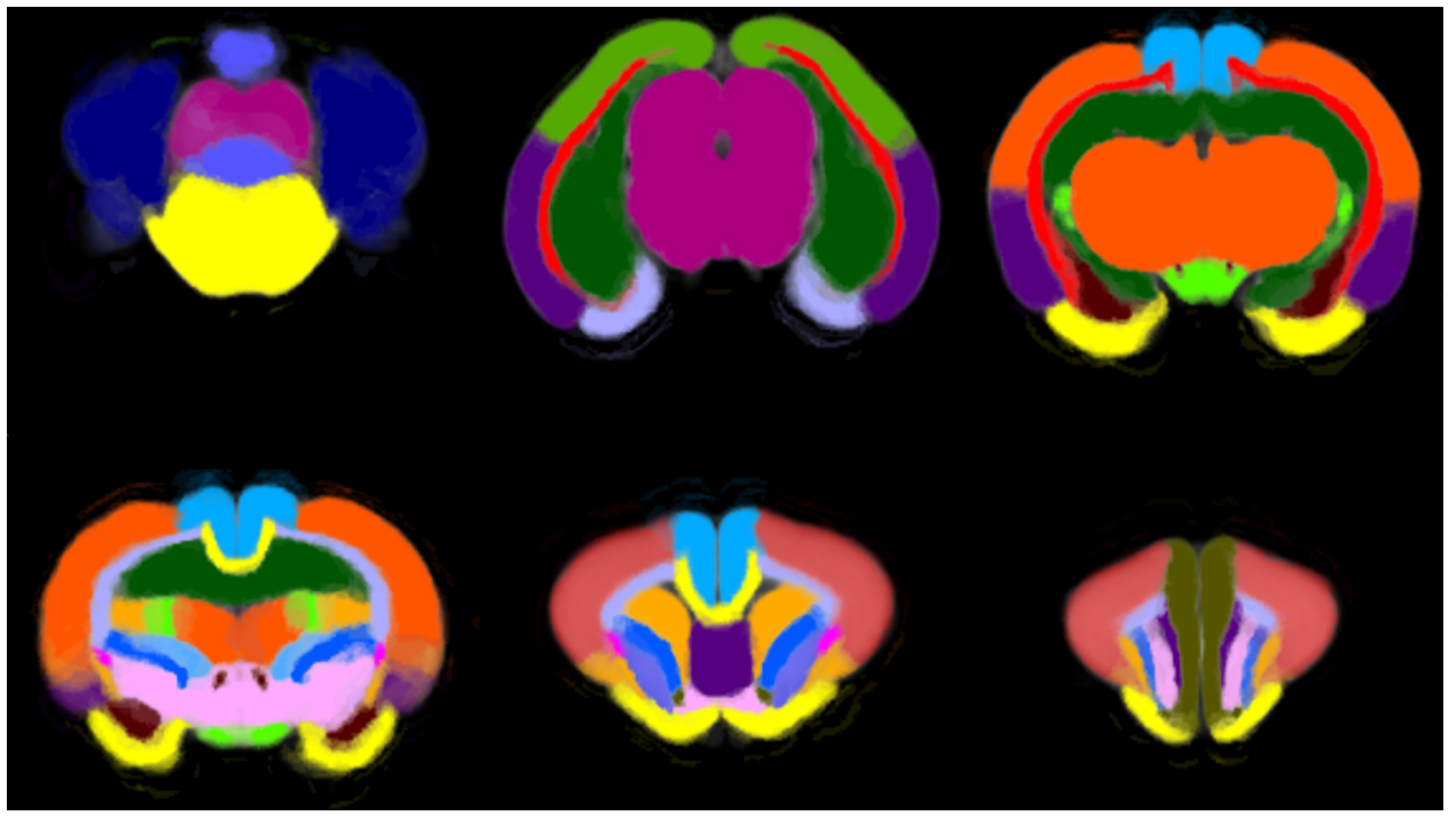
Representative slices of the probabilistic region maps over the template. Color intensity represents the probability value.

Some slices of the template volume are shown in Figure 5. The probability maps of some of the regions are shown in Figure 6, where it can be noted that the contours of regions are fuzzy, since voxels in these areas may belong to neighbor regions.

### Automatic Segmentation

**Figure 7:**
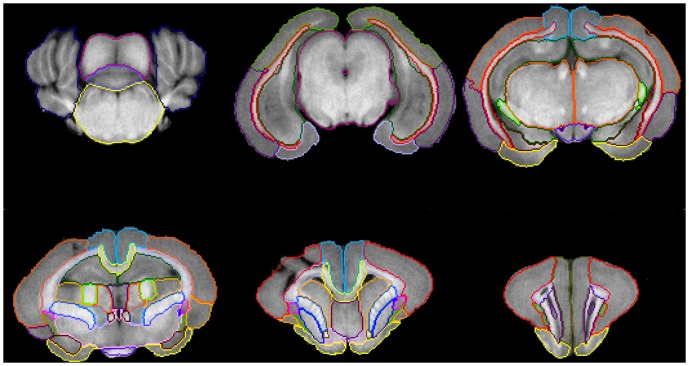
Representative slices of the automatic segmentation of a brain volume.

In order to test the accuracy of segmentation based on the atlas, the brain volume not included in the atlas building was automatically segmented, and compared with the manual delineation of this volume. Segmentation performance was both qualitative and quantitatively evaluated. First, visual inspection of the resulting segmentation confirms appropriate segmentation, as can be viewed in Figure 7, where some slices of the T1-MRI and the overlapped contours of the automatically segmented regions are displayed. It can be observed that the different structures were correctly identified, even in areas where the tissue is broken.

Secondly, objective measures also confirm the similarity between the manual and automatic segmentation: the index for global matching between the automatic segmentation and each of the two manual delineations were 0.9187 and 0.8690; and the Dice coefficient computed between both manual delineation was 0.8779. That is, globally, the accuracy of the automatic parcellation is similar to the accuracy of the manual delineations.

The accuracy of segmentation for each individual region is compiled in Table 2. Note that right and left areas of the same structure are considered as an only region. The three columns in table correspond to: similarity between automatically identified regions and the first manual delineation; similarity between automatically identified regions and the second manual delineation; and similarity between both manual delineations.

**Table 2:** Accuracy of the automatic atlas-based segmentation.

Region	Automatic-Manual 1	Automatic-Manual 2	Interobserver
Frontal cortex	0.8946	0.9003	0.8993
Medial frontal cortex	0.9049	0.8754	0.8442
Cingulate cortex	0.9494	0.8891	0.8737
Piriform cortex	0.7576	0.7874	0.7984
Entorhinal cortex	0.8945	0.8098	0.8681
Parietal cortex	0.8813	0.8847	0.8926
Occipital cortex	0.9437	0.8175	0.8386
Insular cortex	0.7319	0.7366	0.7528
Temporal cortex	0.9849	0.8737	0.8797
External capsule	0.9221	0.7148	0.7051
Internal capsule	0.9522	0.8365	0.8936
Corpus callosum	0.9780	0.8647	0.8895
Anterior commissure	0.8469	0.7109	0.7856
Periventricular white matter	0.9788	0.7666	0.7735
Subcortical white matter	0.7476	0.8359	0.8231
Corona radiata	0.8873	0.7447	0.7547
Fimbria of hippocampus	0.7903	0.7452	0.7191
Fornix	0.8093	0.7286	0.7547
Claustrum	0.9564	0.8845	0.8366
Caudate nucleus	0.9842	0.9399	0.9513
Thalamus	0.9729	0.9012	0.8900
Hippocampus	0.9447	0.7755	0.8575
Amygdala	0.8851	0.8364	0.8577
Hypothalamus	0.7051	0.7695	0.7516
Lenticular nucleus	0.9535	0.7679	0.8513
Olfactory bulb	0.6951	0.7652	0.8916
Cerebellar hemispheres	0.9458	0.7679	0.8513
Vermis	0.9580	0.9112	0.9166
Basal forebrain	0.8664	0.7403	0.7305
Forebrain	0.7573	0.7715	0.7481
Diencephalon	0.7741	0.8231	0.8970
Mesencephalon	0.9636	0.9251	0.9336
Pons	0.9316	0.9255	0.9207
Medulla oblongata	0.9995	0.9368	0.9532
Septum	0.9777	0.9296	0.9074

Dice coefficient between the manually delineated brain regions and the brain regions identified by the automatic atlas-based segmentation, and between the manual delineations performed by two different observers.

**Figure 8:**
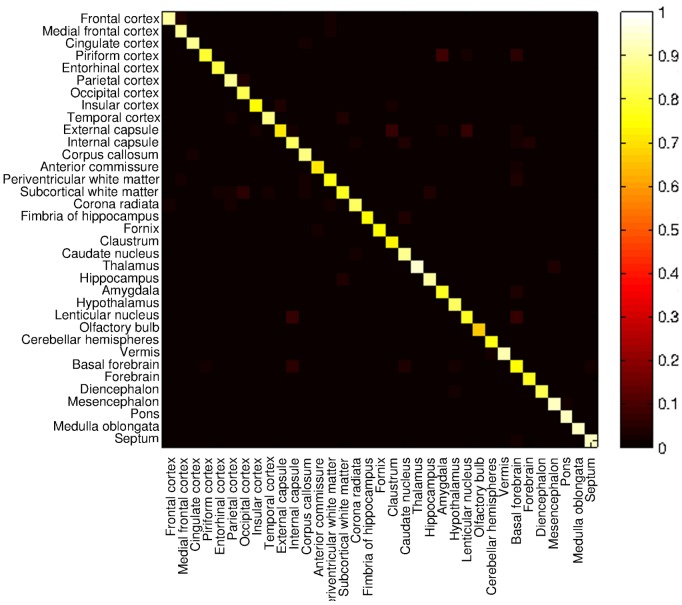
Confusion matrix. Comparison between the automatic segmentation and the manual delineation.

Finally, the confusion matrix is shown in Figure 8. The value at each point 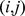 in the matrix is the percentage of voxels belonging to region 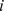 in the manual delineation that have been labeled as region 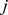 by the automatic segmentation. That is, brighter points corresponds to higher number of points belonging to region 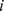 labeled as region 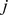. In case of perfect matching, diagonal values would be one (white) and the others point would be zeros. It can be viewed that the resulting confusion matrix for automatic segmentation is close to be diagonal.

## Discussion

### Anatomical Areas Definition

Having an MRI-based rabbit brain atlas to allow automatic segmentation is of great interest since it opens a wide window for neuroimage based analysis as, for instance, connectivity studies. In this regard, manual delineation was performed using both T1-MRI and diffusion MRI data. This multimodal approach allows a more accurate identification of specific structures such as WM tracts. However, the spatial resolution of both types of images limits the delineation of different anatomical structures. For this reason, all structures that could not be delineated were distributed into major divisions of the central nervous system, such as those described in “other regions” category.

Note that delineation was performed over images of post-mortem fixed and excised brains. It must be taken into account that there are morphometric differences between in vivo and in vitro brains [11]. For this reason, special care must be taken if the atlas is applied to segment images of in vivo brains, being necessary appropriate registration algorithms to remove the post-fixation distortion.

### Individual Atlases

Ten individual brain atlases were built in order to avoid the bias due to the choice of a single subject. The low variability among size and intensity values in the ten subjects supports that parcellation of brain regions was highly reproducible.

The delineation scheme here used has been already reported in [10], and simplifies the tedious task of manual delineation. Although it could be argued that a previous automated delineation step can bias the observer, we countered this potential drawback by manual correction verifying the compliance of the delineations with rigorous criteria as assessed by an expert in neuroanatomy.

### Template and Probabilistic Maps

The use of an average model of shape and intensity allows to have a reference template which represents the normal shape and intensity distribution of the rabbit brain, avoiding the inter-subject variability. Probability maps are used in order to deal with the partial volume effect. While individual atlases assign single values to each voxel to identify the region to which the voxel belongs, the probabilistic maps give a probability. This approach may allow a higher accuracy in the definition of region when a new sample image is registered to the template. The number of subjects required to obtain a probabilistic map is not clearly defined. Previous studies suggest that the use of 10 subjects as performed in this study allows to build representative probabilistic atlas [10].

### Automatic Parcellation

In this paper, we have proposed an automatic segmentation method based on the maximization of the region probability at each voxel. To match the template to the data image, a multiresolution block-matching algorithm based on the correlation coefficient between the intensity levels was used. The use of this algorithm allows robust global matching avoiding local minima. However, the atlas here reported could potentially be used with other registration algorithms, such as the implemented in available image processing software.

Quantitative evaluation showed that the differences between the regions automatically and manually identified were comparable to the differences due to the interobserver variability (Table 2), which supports that the atlas can be used for automatic brain parcellation in studies using the rabbit brain. All the regions could be automatically identified by means of registration against the proposed atlas with accuracy values similar to the interobserver differences. It can be noticed that similarity values were higher in bigger regions that in smaller nucleus. In these smaller areas, subtle differences in the contours of the regions have more influence in the final measure of the Dice coefficient, since they represent a higher percentage of all the voxels belonging to the region. For this reason, lower similarity values in these regions were present in the comparison between automatic and manual regions as well as in the comparison between manual delineations. The only region where there was a significant improvement when delineation was performed manually was the olfactory bulb. This fact could be related to the high variability of this structure in our data-set, due to the brain extraction and fixation procedure.

### Conclusions

Atlases have become fundamental in neuroimage, since they are required to identify brain structures in a coherent and objective way in different subjects. Moreover, the use of digital atlases allows automatic segmentation of such structures, avoiding the necessity for manual delineation to perform regional analyses. In this paper, we contribute to solve the lack of digital atlases of the rabbit brain by developing an MRI-based atlas of the New-Zealand rabbit available on line. First, a set of anatomical regions that constitute the rabbit brain have been defined based on the literature. These regions have been identified in a set of ten individuals, showing the reproducibility of the anatomical parcellation in different subjects.

One of the main applications of the anatomical atlas here described is to be used for automatic segmentation. An average template and a probabilistic atlas have been developed from the individual atlases in order to provide a subject-independent reference of brain parcellation and a model of normality for the brain. Moreover, the template and the probabilistic atlases are useful for the development of automatic segmentation algorithms. The ability of the atlas to be used for automatic segmentation has been tested, and the quantitative comparison with manual delineation has shown that similar results are obtained.

Therefore, the atlas here presented will be a useful tool for studies using the rabbit as a model of brain disease.
